# Comparison of setup accuracy and efficiency between the Klarity system and BodyFIX system for spine stereotactic body radiation therapy

**DOI:** 10.1002/acm2.13804

**Published:** 2022-10-09

**Authors:** Enzhuo Quan, Shane P. Krafft, Tina M. Briere, Marissa J. Vaccarelli, Amol J. Ghia, Andrew J. Bishop, Debra N. Yeboa, Todd A. Swanson, Eun Young Han

**Affiliations:** ^1^ Department of Radiation Physics, The University of Texas MD Anderson Cancer Center Houston Texas USA; ^2^ Radiation Oncology The University of Texas MD Anderson Cancer Center Houston Texas USA

**Keywords:** BodyFix, Klarity, spine SBRT

## Abstract

**Background:**

Spine stereotactic body radiation therapy (SBRT) uses highly conformal dose distributions and sharp dose gradients to cover targets in proximity to the spinal cord or cauda equina, which requires precise patient positioning and immobilization to deliver safe treatments.

**Aims:**

Given some limitations with the BodyFIX system in our practice, we sought to evaluate the accuracy and efficiency of the Klarity SBRT patient immobilization system in comparison to the BodyFIX system.

**Methods:**

Twenty‐three patients with 26 metastatic spinal lesions (78 fractions) were enrolled in this prospective observational study with one of two systems – BodyFIX (*n* = 11) or Klarity (*n* = 12). All patients were initially set up to external marks and positioned to match bony anatomy on ExacTrac images. Table corrections given by ExacTrac during setup and intrafractional monitoring and deviations from pre‐ and posttreatment CBCT images were analyzed.

**Results:**

For initial setup accuracy, the Klarity system showed larger differences between initial skin mark alignment and the first bony alignment on ExacTrac than BodyFIX, especially in the vertical (mean [SD] of 5.7 mm [4.1 mm] for Klarity vs. 1.9 mm [1.7 mm] for BodyFIX, *p*‐value < 0.01) and lateral (5.4 mm [5.1 mm] for Klarity vs. 3.2 mm [3.2 mm] for BodyFIX, *p*‐value 0.02) directions. For set‐up stability, no significant differences (all *p*‐values > 0.05) were observed in the maximum magnitude of positional deviations between the two systems. For setup efficiency, Klarity system achieved desired bony alignment with similar number of setup images and similar setup time (14.4 min vs. 15.8 min, *p*‐value = 0.41). For geometric uncertainty, systematic and random errors were found to be slightly less with Klarity than with BodyFIX based on an analytical calculation.

**Conclusion:**

With image‐guided correction of initial alignment by external marks, the Klarity system can provide accurate and efficient patient immobilization. It can be a promising alternative to the BodyFIX system for spine SBRT while providing potential workflow benefits depending on one's practice environment.

## 1 INTRODUCTION

Spine stereotactic body radiation therapy (SBRT) has been shown to be an effective treatment for spine metastases.^1^, ^2^, ^3^, ^4^, ^5^ Given the proximity of target volumes to surrounding organs at risk (OARs), including the spinal cord, spine SBRT requires highly conformal dose distributions with steep dose gradients to achieve adequate tumor coverage and OAR sparing. Highly precise and accurate positioning and immobilization of the patient is crucial to achieve safe and accurate treatment delivery.

The BodyFIX (Elekta, Stockholm, Sweden) stereotactic patient positioning and immobilization system has been used in our practice since 2002 and has produced reproducible, efficient patient setups and favorable treatment outcomes.^5^, ^6^ Similar applications of the BodyFIX system have been reported in the literatures.^7^, ^8^, ^9^, ^10^, ^11^, ^12^, ^13^ The BodyFIX system consists of a full‐body vacuum cushion and a vacuum cover sheet. The vacuum cushion can be indexed to the treatment table, shapes to the contour of the patient, and is the primary source of patient positioning and immobilization. The vacuum cover sheet seals the patient's body within the vacuum cushion and provides additional immobilization support.

While effective, the BodyFIX system did pose several challenges for our expanding spine SBRT program. With an increased use of magnetic resonance (MR) simulation within the spine SBRT practice, it has become clear that the thick vacuum cushion routinely causes clearance issues with the MR bore. There are also challenges associated with safe transportation and storage of these large and often cumbersome vacuum cushions, which prompted us to explore alternative setup and immobilization strategies.

The Klarity SBRT system (Klarity Medical Products, Ohio, US) is one alternative to the BodyFIX system. Instead of relying on large vacuum cushions, the Klarity system uses a full‐length carbon fiber baseplate accompanied by a series of adjustable and indexable accessories to provide reproducible patient positioning and immobilization.

The Klarity SBRT system also utilizes a more open and less restrictive structure, with only a few supporting and restricting positions throughout the body, in contrast to the semi‐rigid full‐body confinement of the BodyFIX system. Although a few studies have shown that less restrictive immobilization devices could produce comparable setup accuracy and stability with the use of image guidance,^14^, ^15^ there have not been published reports on the efficacy of the Klarity system for SBRT at any site, or a comparison with the BodyFIX system. In this study, we used the patient setup data collected from an internal review board (IRB)‐approved prospective clinical trial in our institution to thoroughly evaluate the Klarity SBRT system in comparison to the BodyFIX system for spine SBRT. The goal of this study is to assess its setup accuracy, setup efficiency, and intrafractional position stability.

## 2 METHOD

### 2.1 Patient selection

Twenty‐three patients were enrolled in an internal study (IRB PA2018‐1096). Each patient was simulated and treated using one of two immobilization systems (BodyFIX and Klarity SBRT systems). Lesions in the C‐spine and upper T‐spine were excluded in this study because the setup uses a 5‐point mask that is essentially the same for both SBRT systems.

### 2.2 Patient simulation

**FIGURE 1:**
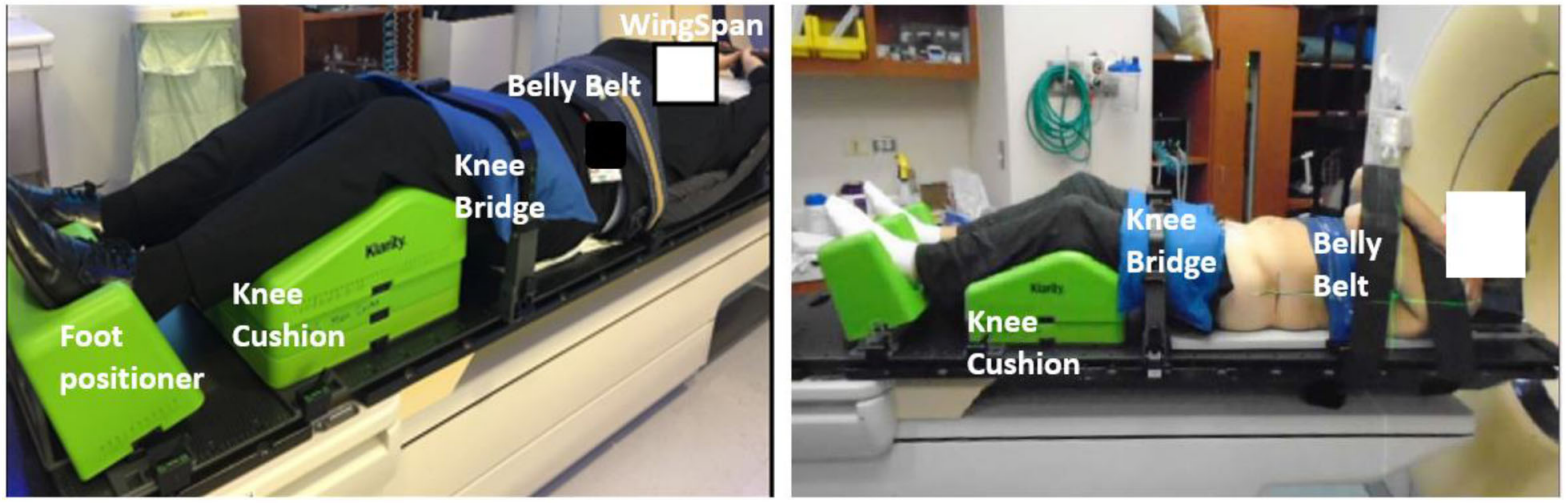
Patient setup examples with Klarity stereotactic body radiation therapy (SBRT) system with arms up in cradle (T spine, left) and crossed on chest (L‐S spine, right)

All patients were simulated in a supine position with arms being either “up” (above the shoulders) or “down” (crossed on the chest or straight on the sides of the body). For BodyFIX, the patient's entire body was supported by the full‐body vacuum cushion, and a vacuumed plastic sheet covered the patient's body from neck to feet. For Klarity, a 100 cm^2^ vacuum cushion placed over the WingSpan baseplate supported the patient's arms for the “Arms Up” setup, and straps were used to support the arms for the “Arms Down” setup. Additionally, a custom‐designed belly belt (not used for respiratory compression) and a knee bridge with a small anterior vacuum bag were used for all patients. Anterior isocenter marks were placed on the skin for all patients; lateral isocenter marks were placed on the vacuum cushion for the BodyFIX system and on the skin for the Klarity system. Two examples of patient setups with the Klarity system are shown in Figure 1. Treatment planning was done in either Pinnacle (Philips Radiation Oncology Systems, Fitchburg, WI, USA) or RayStation (RaySearch, Stockholm, Sweden) with four posteriorly directed partial arcs or nine posterior oblique intensity‐modulated radiation therapy (IMRT) beams.

### 2.3 SBRT treatment workflow

All treatments were delivered on a Varian Truebeam equipped with a high‐definition multileaf collimator, a 6 degrees of freedom (DoFs) couch and the ExacTrac (Brainlab Inc., Westchester, IL, USA) imaging system. Patients were initially set up to marks on the cradle and/or skin that were placed at simulation and then manually shifted to final isocenter. ExacTrac images were subsequently taken for initial image‐guided radiation therapy positioning; if initial setup tolerances (0.7 mm/0.8°) were exceeded after initial couch shifts, additional ExacTrac images were acquired, and further table corrections were applied. When satisfactory patient alignment was achieved based on ExacTrac, a pre‐treatment cone‐beam computed tomography (CBCT) was taken to serve as 3D position verification, and a kV/MV orthogonal pair of images was acquired to confirm gross vertebrae alignment. During treatment, monoscopic X‐rays were taken to monitor intrafractional patient motion; if the patient motion was detected, stereoscopic X‐rays were then taken to evaluate and correct the patient motion as necessary.

### 2.4 Assessments of setup accuracy, setup efficiency, intrafractional position stability

The magnitude of initial corrections in 6DoFs was compared between the BodyFIX and Klarity systems. The maximum table deviations given by series of ExacTrac images taken for the initial table correction during the setup procedure were also compared (setup accuracy). Similarly, comparisons of maximum deviations during treatment were performed for the assessment of intrafractional position stability. For setup efficiency assessment, we recorded the total number of ExacTrac and CBCT images taken during the setup procedure and the number of table corrections that were applied. The total setup time was calculated as the time from the first ExacTrac image to the start of the first beam; the total treatment time was calculated as the time from the start of the first beam to the end of the last beam minus beam‐on time, which corresponds to the total image‐guidance time during each treatment. Statistical analyses were performed using the unpaired, two‐tailed *t*‐test, and a *p*‐value of less than 0.05 was considered statistically significant.

### 2.5 Assessment of geometric uncertainty

For a subset of patient treatments (7 lesions with BodyFIX and 11 lesions with Klarity), an additional planned CBCT image set was acquired at the end of the treatment (posttreatment CBCT). Each patient's last CBCT acquired before treatment (pre‐CBCT) and posttreatment CBCT (post‐CBCT) were rigidly registered with the planning CT that was acquired at simulation (p‐CT) based on bony anatomy near the target region.

For each set of registrations, the translational (vertical, longitudinal, and lateral) and rotational (yaw, pitch, and roll) deviations were recorded at the treatment console. The geometric uncertainty of the two immobilization systems was calculated based on each of the deviations.^16^ The geometric uncertainty is stratified by systematic error (*Σ*) and random error (*σ*); both *Σ* and *σ* include errors from the initial setup (s) and the intrafractional motion (m):

(1) $$\sum =\sum_{s}+\sum_{m},\;\mathrm{and}\;\sigma =\sigma_{s}+\sigma_{m}.$$

The initial setup errors were calculated based on the deviations between the pre‐CBCT, and p‐CT and the intrafractional motion errors were calculated based on the deviations between the post‐CBCT and pre‐CBCT.

## 3 RESULTS

### 3.1 Patient Characteristics

Twenty‐three patients with 26 metastatic spinal lesions (78 fractions) were analyzed. Eleven patients (14 lesions) were treated using the BodyFIX system and 12 patients (12 lesions) with Klarity, as listed in Table 1. Lesions were distributed in the lower T‐spine to the acetabulum, with the most common vertebral body being thoracic spine (54%) followed by lumbar spine (38%). The median number of fractions was 3 (92%, range 1–5).

**TABLE 1:** Lesion locations and fractions for patients simulated with BodyFIX and Klarity systems

BodyFIX				Klarity			
Lesion (patient)	Arm position	Site	Number of fractions	Lesion (patient)	Arm position	Site	Number of fractions
1 (1) *	down	T4‐5	3	1 (1) *	down	T8	3
2 (2) *	up	T5	3	2 (2) *	up	T8	3
3 (3) *	down	T6‐7	3	3 (3) *	up	T8‐T9	3
4 (4)	up	T6‐7	3	4 (4) *	down	T11‐T12	3
5 (1) *	down	T7	3	5 (5) *	down	T12‐L3	3
6 (5)	down	T7‐8	3	6 (6) *	down	L1	3
7 (6)	down	T7‐10	3	7 (7) *	down	L2‐L4	3
8 (4) *	up	T9	3	8 (8)	down	L4	3
9 (7) *	down	T9‐10	3	9 (9) *	down	L4‐S2	3
10 (8) *	down	T10	3	10 (10) *	down	L5	3
11 (9)	down	L1	3	11 (11) *	down	L5‐S2	3
12 (10)	down	L4	3	12 (12) *	down	S1‐S3	3
13 (10)	down	L4	1				
14 (9)	down	Rt Acetab	5				

* Lesions with posttreatment CBCT taken for geometric uncertainty analysis.

### 3.2 Assessment of setup accuracy

**FIGURE 2:**
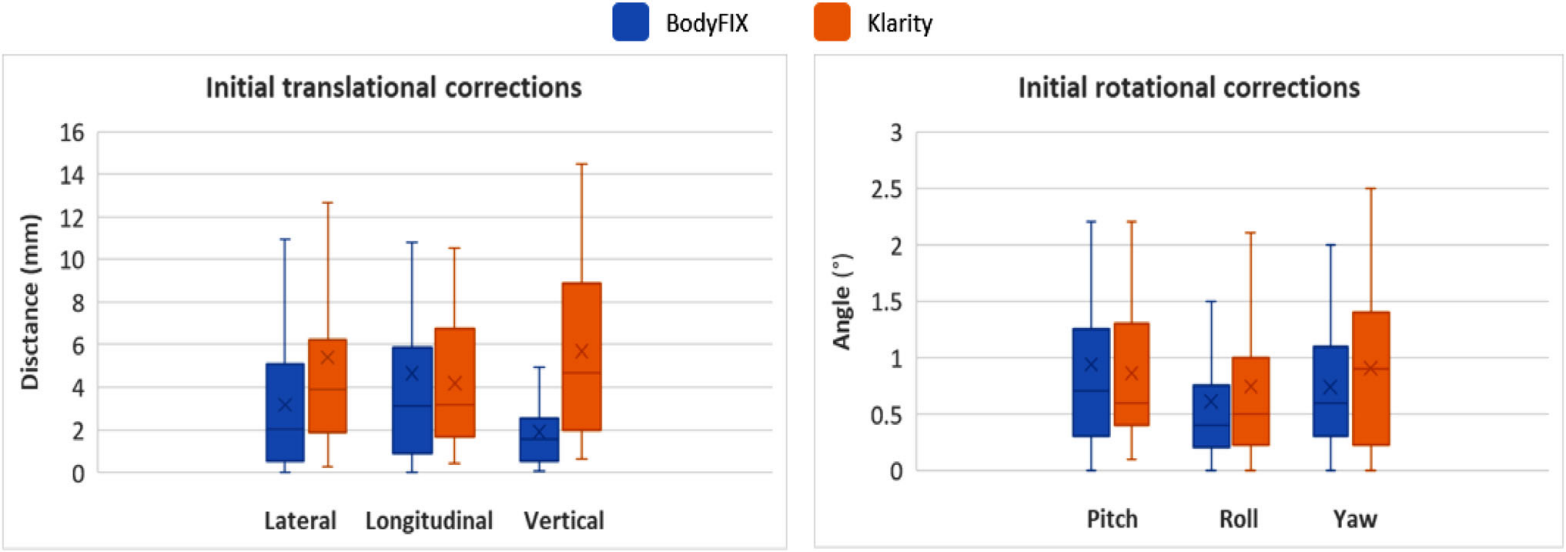
Initial ExacTrac table corrections in the translational and rotational directions for the BodyFIX and Klarity systems

The two immobilization systems showed similar magnitude in the table corrections along the longitudinal and three rotational directions in the initial ExacTrac‐based table corrections after skin‐mark alignment (all *p*‐values > 0.05). However, in the lateral and vertical directions, the Klarity system had larger corrections compared to the BodyFIX system (Figure 2 and Table 2, Section I) – a mean lateral correction of 3.2 mm for BodyFIX versus 5.4 mm for Klarity (*p*‐value = 0.02) and a mean vertical correction of 1.9 mm for BodyFIX versus 5.7 mm for Klarity (*p*‐value < 0.01).

**TABLE 2:** Initial table corrections, maximum position deviations during setup, and maximum position deviations during treatment

Axis	*Section I*			*Section II*			*Section III*		
	Initial corrections (Mean ± SD)			Max deviations during setup (Mean ± SD)			Max deviations during treatment (Mean ± SD)		
	BodyFIX	Klarity	*p*‐Value	BodyFIX	Klarity	*p*‐Value	BodyFIX	Klarity	*p*‐Value
Lateral (mm)	**3.2 ± 3.2**	**5.4 ± 5.1**	**0.02**	0.7 ± 1.2	1.0 ± 1.0	0.79	0.5 ± 0.6	0.6 ± 0.6	0.56
Longitudinal (mm)	4.6 ± 6.5	4.2 ± 3.1	0.69	0.6 ± 0.7	0.7 ± 0.5	0.82	0.4 ± 0.4	0.5 ± 0.3	0.17
Vertical (mm)	**1.9 ± 1.7**	**5.7 ± 4.1**	**<0.01**	0.6 ± 1.4	0.5 ± 0.6	0.82	0.5 ± 0.4	0.3 ± 0.3	0.26
Pitch (°)	0.9 ± 0.9	0.9 ± 0.7	0.66	0.5 ± 0.4	0.5 ± 0.5	0.52	0.4 ± 0.4	0.3 ± 0.2	0.63
Roll (°)	0.6 ± 0.6	0.7 ± 0.7	0.37	0.3 ± 0.2	0.4 ± 0.4	0.94	0.3 ± 0.2	0.3 ± 0.3	0.73
Yaw (°)	0.7 ± 0.6	0.9 ± 0.7	0.24	0.3 ± 0.3	0.3 ± 0.2	0.24	0.3 ± 0.2	0.3 ± 0.2	0.56

* Statistically significant differences (*p*‐value < 0.05) are highlighted. SD‐ Standard Deviation.

**FIGURE 3:**
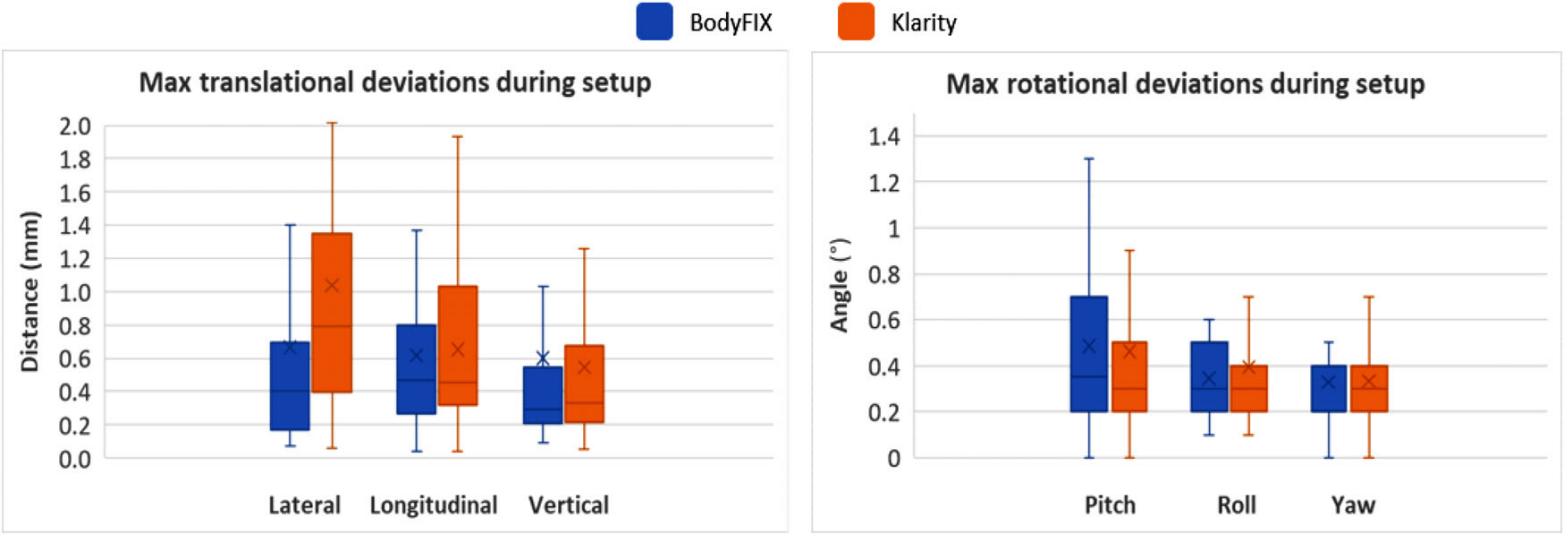
Maximum position deviations during setup in the translational and rotational directions for the BodyFIX and Klarity systems

Importantly, in the subsequent image‐guided setup procedure after the first table corrections were applied, no significant differences (all *p*‐values > 0.05) were observed in the maximum magnitude of positional deviations between the two systems, as shown in Figure 3 and Table 2 (Section II). Mean translational deviations were ≤1.0 mm and all rotational deviations ≤0.5°.

### 3.3 Assessment of setup efficiency

The Klarity system required an average of 1.6 additional table corrections during setup to keep the patient within tolerance, whereas the BodyFIX system required an average of 0.8 additional table corrections (*p*‐value = 0.01), as shown in Table 3. However, both systems achieved desired bony‐alignment with similar total numbers of ExacTrac images (4.1 for BodyFIX and 4.5 for Klarity, *p*‐value = 0.56) and CBCT images (1.2 for BodyFIX and 1.4 for Klarity, *p*‐value = 0.06) and similar total setup time (15.8 min for BodyFIX and 14.4 min for Klarity, *p*‐value = 0.41), as shown in Table 3.

**TABLE 3:** Setup efficiency comparisons between the two immobilization systems

	Number of ExacTrac images acquired during setup	Number of table corrections made during setup	Number of CBCT images acquired during setup	Setup time (min)	Treatment time (excluding beam‐on time) (min)
BodyFIX	4.1 ± 2.4	**0.8 ± 1.2**	1.2 ± 0.4	15.8 ± 8.0	7.6 ± 4.7
Klarity	4.5 ± 2.6	**1.6 ± 1.4**	1.4 ± 0.7	14.4 ± 7.6	8.3 ± 4.2
*p*‐value	0.56	**0.01**	0.06	0.41	0.48

### 3.4 Assessment of intrafractional position stability

**FIGURE 4:**
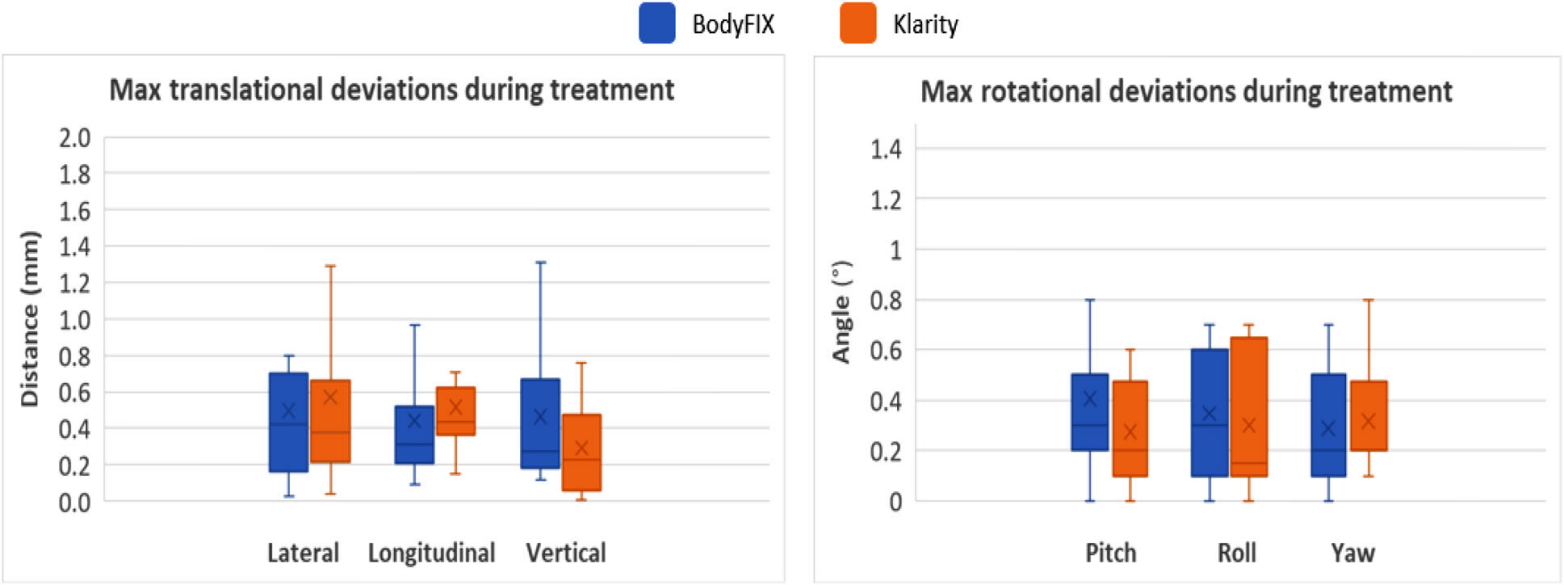
Maximum position deviations during treatment in the translational and rotational directions for the Klarity and BodyFIX systems

During patient treatment, 24 of 36 fractions of Klarity setups (67%) and 23 of 42 fractions of BodyFIX setups (55%) required only monoscopic X‐rays during intrafractional ExacTrac monitoring. In the remaining fractions, from the table position deviations given by stereoscopic X‐rays, the two systems did not show significant differences in any of the translational or rotational directions, with translational deviations ≤0.6 mm and rotational deviations ≤0.4° (all *p*‐values > 0.05), as shown in Figure 4 and Table 2 (Section III). In addition, treatment times (excluding beam‐on time) were similar for the two systems (Table 3, last column) – 7.6 min for BodyFIX and 8.3 min for Klarity (*p*‐value = 0.48).

### 3.5 Assessment of geometric uncertainty

Table 4 provides systematic and random errors for all six translational and rotational directions for the two systems based on registrations from the pre‐ and posttreatment positions (pre‐CBCT and post‐CBCT) to the simulation position (p‐CT). The Klarity system resulted in similar geometric uncertainty in both systematic errors and random errors in vertical direction and pitch, and random error in yaw, but slightly smaller geometric uncertainty in other directions than the BodyFIX system as shown in Table 4.

**TABLE 4:** Geometric uncertainties of the BodyFIX and Klarity systems calculated from pre‐ and posttreatment CBCT deviations

**BodyFIX system**	Translation (mm)			Rotation (°)		
	Vertical	Longitudinal	Lateral	Yaw	Pitch	Roll
Systematic error	0.4	0.6	0.6	0.4	0.4	0.5
Random error	0.4	0.9	0.8	0.4	0.4	0.4
**Klarity system**						
Systematic error	0.4	0.5	0.4	0.2	0.4	0.2
Random error	0.4	0.5	0.7	0.4	0.4	0.3

## 4 DISCUSSION

This is the first known study to compare the performance, as measured by accuracy and efficiency, between two immobilization systems for spinal SBRT treatment. As previously reported, the BodyFIX system has been widely used.^7^, ^9^, ^11^, ^13^, ^17^, ^18^ However, the newer Klarity system has less published performance data. Based on our study, the Klarity system provides a reproducible and efficient setup for spine SBRT. Additionally, as the first study to thoroughly evaluate the Klarity SBRT system in comparison to the BodyFIX system for the use in spine SBRT, we found comparable results.

The challenges posed by using BodyFIX such as issues related to the MRI, transportation and storage have been drastically mitigated by changing to the Klarity system even though those were not quantified in this study. Given these workflow benefits, our findings that the overall setup accuracy and efficiency of the Klarity system is similar to the BodyFIX system are encouraging.

The initial setup accuracy revealed that the BodyFIX system permitted slightly more reliable setup in the lateral and vertical directions and performed similarly to Klarity in other directions. This is likely because of the tighter enclosure of the BlueBAG vacuum cushion around the patient's body laterally and posteriorly than the Klarity baseplate.

Comparing the setup accuracy and intrafractional motion between the two systems, the analysis of ExacTrac registration results did not show a statistically significant difference in any direction. The setup accuracy was also evaluated using the registration of CBCT images acquired immediately before and after beam delivery with planning CT images, which showed that the Klarity system results in slightly reduced geometric uncertainty in almost all directions compared to the BodyFIX system.

A few factors may have contributed to the Klarity system's reduced geometric uncertainty from this calculation. The number of CBCT image sets used in this calculation was 7 for the BodyFIX system and 12 for the Klarity system. The fewer number of image sets for the BodyFIX system might have contributed to the larger calculated uncertainty. On the other hand, the design of the Klarity system may have the potential to improve positioning accuracy with its adjustable and indexable accessories. For example, the knee wedge has three different height options and can be indexed at any position on the baseplate based on patient comfort; at the treatment setup, it is set to the same height and position as recorded during simulation, which helps to accurately re‐produce the patient's lower body position and hence the curvature in the spine.

The two systems were comparable in the number of ExacTrac and CBCT images acquired during setup, total setup time, and total treatment time. The only exception is that slightly more table corrections were needed during initial setup with the Klarity system than the BodyFIX system to meet the tolerances. This is likely a consequence of the larger initial table corrections due to the less accurate skin‐mark‐based alignment – 24 of the 36 fractions of Klarity setups required a second table correction immediately following the initial correction, whereas only 5 of the 42 fractions of the BodyFIX setups required a second table correction. However, this did not appear to have affected the overall setup time or setup accuracy.

The results of this study should be interpreted with the following limitations in mind. Although patients with lesions located at various vertebral levels were involved in this study, a larger and randomly selected patient sample may further reduce study bias and improve the statistical significance of the study. If the two types of immobilization systems could be applied to the same subject, the bias caused by individual differences in the selected patients could be ruled out; however, this was not achievable due to clinical restrictions.

## 5 CONCLUSION

With image‐guided correction of initial alignment, the Klarity system can provide accurate and efficient patient immobilization. Although larger vertical and lateral initial table corrections were needed with the Klarity system, the overall setup accuracy, setup efficiency, and intra‐fractional stability were comparable to the BodyFIX system. Therefore, this system is a promising alternative to the BodyFIX system for Spine SBRT while providing potential workflow benefits depending on one's practice environment.

## CONFLICT OF INTEREST

The authors declare that there is no conflict of interest that could be perceived as prejudicing the impartiality of the research reported.
